# RM-SORN: a reward-modulated self-organizing recurrent neural network

**DOI:** 10.3389/fncom.2015.00036

**Published:** 2015-03-24

**Authors:** Witali Aswolinskiy, Gordon Pipa

**Affiliations:** Institute of Cognitive Science, University of OsnabrückOsnabrück, Germany

**Keywords:** reward-modulated STDP, intrinsic plasticity, recurrent neural networks, self-organization, plasticity, hebbian learning

## Abstract

Neural plasticity plays an important role in learning and memory. Reward-modulation of plasticity offers an explanation for the ability of the brain to adapt its neural activity to achieve a rewarded goal. Here, we define a neural network model that learns through the interaction of Intrinsic Plasticity (IP) and reward-modulated Spike-Timing-Dependent Plasticity (STDP). IP enables the network to explore possible output sequences and STDP, modulated by reward, reinforces the creation of the rewarded output sequences. The model is tested on tasks for prediction, recall, non-linear computation, pattern recognition, and sequence generation. It achieves performance comparable to networks trained with supervised learning, while using simple, biologically motivated plasticity rules, and rewarding strategies. The results confirm the importance of investigating the interaction of several plasticity rules in the context of reward-modulated learning and whether reward-modulated self-organization can explain the amazing capabilities of the brain.

## Introduction

The brain is a complex, self-organizing system, where a multitude of neural plasticity mechanisms shape learning, and memory. These plasticity mechanisms are, in turn, shaped by neuromodulators, which are often part of a reward system (Pawlak et al., 2010). *In vivo* experiments showed that rewarding behavior can change synapses and neurons selectively to achieve a rewarded goal (Fetz, 1969; Ahissar et al., 1992; Sigala and Logothetis, 2002). Several models of reward-modulated recurrent neural networks are able to partially replicate these experiments and solve simple tasks (Izhikevich, 2007; Legenstein et al., 2008; Soltoggio and Steil, 2013; Hoerzer et al., 2014). In these models, correct outputs are rewarded through the application of STDP or a hebbian learning rule, and noise is used to explore possible output sequences. Noise as a part of a model, however, makes the model non-deterministic and introduces a random, transient component that can counteract the learning of causal relations by STDP. Here, we propose an alternative to combine deterministic behavior and the ability to explore states for reward modulated learning. For this, either deterministic chaos or other complex deterministic behavior may be used. Here, we study complex behavior that is introduced by Intrinsic Plasticity (IP)—neuronal plasticity associated with homeostasis (Turrigiano et al., 1998; Desai et al., 1999). We introduce a simple binary neural network model, which learns through interaction of IP and reward-modulated STDP. Exploration of the output state space is carried out through IP and not noise.

The Reward-Modulated Self-Organizing Recurrent Network (RM-SORN), model is based on SORN: Self-Organizing Recurrent Network (Lazar et al., 2009). SORN consists of a recurrent layer with binary thresholded neurons and a readout layer. In the recurrent layer three plasticity mechanisms are applied: IP, Synaptic Normalization (SN) and Spike-Timing-Dependent Plasticity (STDP). The readout layer is trained with linear regression. The network is trained in two phases: in the first phase the recurrent layer processes the input with ongoing plasticity. In the second phase plasticity is disabled, the recurrent layer processes the input again, and the neuron activations serve to train the readout layer. Lazar showed, that all three plasticity mechanisms are necessary to create an effective representation of the input in the recurrent layer and that these representations allow SORN to outperform randomly initialized non-plastic networks. STDP forms the internal representations, IP activates silent neurons and dampens neurons with too high activity, and SN decorrelates neurons preventing seizure-like activity.

SORN was successfully applied to tasks for prediction (Lazar et al., 2009), recall and non-linear computation (Toutounji and Pipa, 2014) and artificial grammar learning (Duarte et al., 2014). The main advantage of SORN is it's simplicity and the biological plausibility of the plastic recurrent layer. The biological plausibility is further underlined by the findings of Zheng et al. (2013), who added two plasticity mechanisms to the recurrent layer: structural plasticity and inhibitive STDP. The authors observed a log-normal weight distribution of the synaptic weights in the recurrent layer matching experimental findings. Additionally, the patterns of fluctuation of the weights were consistent with the dynamics of dendritic spines found in rat hippocampus.

SORN offers the possibility to study plasticity mechanisms similar to those in the brain in simple, manageable networks. The biologically not plausible part of SORN is the linear regression readout, which is replaced here by a plastic, non-recurrent, reward-modulated neuron layer.

## Materials and methods

Both SORN and RM-SORN consist of a recurrent layer with three plasticity mechanisms and a readout or output layer. However, whereas in SORN the output is trained with linear regression, in RM-SORN, the weights from the recurrent layer to the output layer are plastic and adapted through reward-modulated STDP. The model allows, but doesn't prescribe, the application of reward-modulated STDP to the recurrent layer. In this paper, we test both versions, i.e., with and without reward-modulated STDP in the recurrent network, and explain for which conditions reward-modulated STDP applied to the weights in the recurrent network improves the computational performance.

### Network model

**Figure 1:**
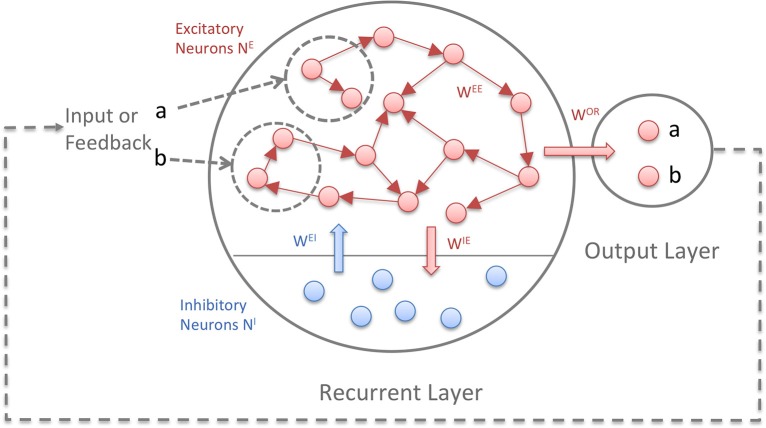
**The Reward-Modulated Self-Organizing Recurrent Neural Network (RM-SORN)**. Excitatory units are depicted as red, inhibitory units as blue. The arrows symbolize directed connections between units. Excitatory units are sparsely interconnected and excitatory and inhibitory units are fully connected between each other. Only the excitatory units project to the output layer. Input and output are sequences of predefined symbols.

Figure 1 depicts the model structure. Both layers consist of binary threshold neurons. The first layer is recurrent and consists of *N^E^* excitatory and *N^I^* inhibitory neurons. The connectivity between the excitatory neurons is sparse (5–10%) and full between excitatory and inhibitory neurons. Self-connections are not allowed. The ratio of excitatory to inhibitory neurons is 5:1. The second layer is the output or readout layer with neurons that are not interconnected. In tasks where the network has to generate sequences, a feedback connection from the output layer to the recurrent layer is necessary. A random subset of the excitatory units in the recurrent layer receives the input: for each symbol of an input sequence, e.g., “1234,” a different subset of the units receives an input of 1 and the rest 0. The units are binary with Θ being the heaviside step function, applied independently to every neuron:

(1) $$\begin{array}{l} x_{i}(t+1)=\Theta (\sum_{j}^{N^{E}}w_{ij}^{EE}x_{j}(t)-\sum_{k}^{N^{I}}w_{ik}^{EI}y_{k}\left(t\right)\\ \;+u_{i}\left(t\right)-\;T_{i}^{E}(t)) \end{array}$$

(2) $$y_{i}\left(t+1\right)=\Theta \left(\sum_{j}^{N^{E}}w_{ij}^{IE}x_{j}\left(t\right)-T_{i}^{I}\right)$$

(3) $$o_{i}\left(t+1\right)=\text{a}\left(\sum_{j}^{N^{E}}w_{ij}^{OE}x_{j}(t)-T_{i}^{O}(t)\right)$$

Neurons in the first layer are updated according to (1) and (2) while neurons in the output layer are updated according to (3). *x* and *y* represent the activity of the excitatory and inhibitory neurons in the recurrent layer and *o* the activity of the output neurons. *a* is the activation function for the output neurons. With several output neurons, the activation function is winner-takes-all (WTA), with one output neuron, the heaviside step function Θ is used. *w^AB^* is the weight matrix with weights from B-units to A-units, *u* is the input and *T^A^* is the threshold for the A-units. (Hence, *w^OE^* are the weights from the recurrent to the output layer and *T^O^* are the thresholds for the output neurons). Initially, the weights are drawn from a normal distribution, but change through application of three plasticity rules: IP, SN, and reward-modulated Spike-Timing-Dependent Plasticity (rm-STDP).

### Plasticity rules

IP adapts the thresholds so that on average each neuron has the firing rate μ*_IP_*:

(4) $$△T_{i}^{E}\left(t\right)\;=\eta_{IP}\left(x_{i}\left(t\right)-\mu_{IP}\right)$$

The threshold is increased when the unit was active and decreased when the unit was inactive, leading to an asymptotic fix point of the average firing rate μ*_IP_*. Thereby, IP activates neurons, which would be otherwise inactive and regulates down neurons which fire too often, enforcing the given average firing rate. During the experiments, in the recurrent layer, μ*_IP_* was set to values between 0.05 and 0.25 depending on which value performed best. In the output layer, μ*_IP_* was set per neuron to correspond to the expected occurrence probability of the symbol represented by the neuron. η*_IP_* is the learning rate for IP.

STDP strengthens the connection from *x_j_* to *x_i_* when *x_j_* was active before *x_i_* (*x_j_* “causes” *x_i_*) and weakens it, when *x_j_* was active after *x_i_*. The main difference to SORN is the modeling of the output layer as another plastic neuron layer and the reward-modulation of STDP with the modulation *m*:

(5) $$△w_{ij}^{EE}=m_{r}*\eta_{STDP}\left(x_{j}\left(t-1\right)x_{i}\left(t\right)-x_{j}\left(t\right)x_{i}\left(t-1\right)\right)$$

(6) $$△w_{ij}^{OE}=m_{o}*\eta_{STDP}\left(x_{j}\left(t-1\right)o_{i}\left(t\right)\right)$$

η_STDP_ is the learning rate for STDP. *m_r_* and *m_o_* are the modulation factors for the recurrent and the output layer, respectively. During the simulations, *m_r_* was either set to one (no modulation) or to the same value as *m_o_*. *m_o_* is determined according to a rewarding strategy, which is a function of the reward *r*. Both modulation and reward can be positive, negative or zero. Depending on the task, different modulation strategies can be chosen for *m_o_*.

After application of STDP the incoming weights to a neuron are scaled to sum up to 1:

(7) $$w_{ij}\left(t\right)\;=\frac{w_{ij}\left(t\right)}{\sum_{j}w_{ij}\left(t\right)}\to \sum_{j}w_{ij}\left(t\right)\;=1$$

The relative strength of the synapses remains the same.

### Reward-modulation strategies

In tasks with known target values (which are all tasks except the generation task), the reward was set to 1 for correct outputs, and either 0 or −1 for wrong outputs, depending on which setting led to the highest performance. Negative reward—punishment—can lead to Anti-STDP. In the generation task, where the network has to generate a sequence of symbols without input, the network is rewarded if it produces a part of the target sequence, starting from the beginning of the sequence. The reward is proportional to the length of the correctly generated sequence part. For example, let the network be rewarded for generation of the sequence “1234.” If the network generates “1234,” it receives the full reward of unit 1 at the time when it generates the last state “4.” Analogous, it receives the reward of ¾ for the sequence “x123,” 2/4 for the sequence “xx12” and only ¼ for “xxx1” (here “x” represents any symbol or state). An exception is made for “xx11”: this combination is punished to prevent the generation of the trivial sequence of repetitions of “1.” For any other sequence, no reward is given.

The reward-modulation strategy (*M*) determines the modulation factor *m* and therefore, whether STDP is applied, suppressed or inversed. The network can be modulated either directly by (8) or the modulation can be computed from the previous rewards. Particularly interesting is here the hypothesis, that dopamine neurons encode reward prediction errors. (9) defines a simple estimate of the reward prediction error.

(8) M0: m(r, t)=r(t)

(9) $$Mk:\;m\left(r,\;k,\;t\right)=r\left(t\right)\;-\;\bar{r}\left(t,\;k\right),\;k\in \left(1,\;5,\;10,\;20\right)$$

*r* is computed as the moving average of the previous *k* rewards. With window size *k* = 1, the modulation factor is the current reward minus the previous reward. The *k*-values were defined *ad-hoc* and selected for each task independently with a parameter grid search. The selected strategies for the tasks and other parameters are listed in the supplementary material.

### Training and testing

The network is trained in two phases:

1. In tasks with training data, the network is reward-modulated, while processing 20,000 steps of training data. In the generation task, the network output is fed back as input. Every 100 steps, the network is validated on 500 steps of validation data. After 20,000 steps, 200 networks are evaluated and the network with the best validation performance is chosen for the next phase.
2. Step 1 is repeated while the plasticity in the first layer is shut off, allowing to fine-tune the weights to the output layer.

After this composed training phase, the network is tested on 10,000 steps of test data or, in generation tasks, is used as a generative model to generate the desired output for 10,000 steps.

The evaluation of the network at different plasticity times is essential, since the performance of the networks fluctuates strongly during reward-modulated plasticity.

During the simulations, the network size consisted of at least 100 and at most 400 excitatory neurons. The number of inhibitory neurons was always a fifth of the excitatory neurons, as in the original SORN. From here on, the number of excitatory neurons will be referred to as the size of the network or simply *N*.

### Model evaluation

The performance of the model was compared to SORN, static (non-plastic) supervised trained networks and random networks. In SORN networks, the 20,000 steps of training data were processed by the plastic recurrent layer. After every 1000 steps, the weights were frozen, the network processed the 20,000 steps of training data and the readout weights were trained on the resulting network states. Thus, 20 intermediary networks were created, with the first network being subjected to plasticity for 1000 steps and the last network for 20,000 steps. The network that performed best on 500 steps of validation data was chosen for the performance evaluation on the test data. Static networks were created by taking the best SORN network, shuffling the weights 20 times and choosing the network that performed best on the validation data. Random networks were trained in the same manner as RM-SORN, but with the reward-modulated STDP-weight updates randomly distributed across all eligible weights at each step. The eligible weights in the recurrent layer are the initially non-zero weights in the sparse connectivity matrix. (Initially non-zero weights can become zero through STDP). From the recurrent layer to the output layer all weights are eligible. The performance difference between the RM-SORN and “random” networks is the difference between learning through reward-modulated STDP and lucky guessing.

Notably, during training of a network, 200 RM-SORN, but only 20 SORN and static-networks were evaluated. This discrepancy is due to the high computational cost of the linear regression in SORN and the fact, that SORN achieves in most tasks almost perfect performance - more frequent evaluation is not necessary. In the pattern recognition task, where RM-SORN was better, the same number of networks (200) was evaluated to even the odds.

All results were averaged over ten data sets and ten networks per data set—the procedure described above was applied to each of the 100 network/data set combinations. In the motion generation task, which has no input, 100 networks were evaluated.

Since the weights in RM-SORN are positive, a similar restriction was imposed onto the supervised training—instead of least squares, non-negative least squares method (Lawson and Hanson, 1995) was used.

### Task descriptions

The model was evaluated on eight different tasks, including those in Lazar et al. (2009) and Toutounji and Pipa (2014).

In the **counting** task (Lazar et al., 2009), the network receives random alternations of two words “abbb…c” and “eddd…f” with *n* + 2 letters per word and n b's or n d's in a word. The goal is to predict the next letter. In order to correctly predict the *last* letter in the word, the network has to “count” the b's and the d's. Thus, the first layer needs to learn separable representations (linearly separable when using a simple linear readout, as we use here) of the input conditions a, b_1_, b_2_,… b*_n_*, e, d_1_, d_2_,…, d*_n_*. In the output layer, these representations must be mapped to the next letter in the word: a→b, b_1_→ b, b_2_ → b… b_n − 1_ → b b_n_ → c and similar for e's and d's. Given the random alternation of words, the first letter of a word is unpredictable and therefore excluded from the performance measure. We use two kinds of performance measures. Firstly, the overall performance that measures the match of all letters of the entire sequence, with the exception of the excluded first letter of each word. Secondly, we measure the counting performance that is the accuracy of predicting the last letter in a word. This performance reflects the capability of the network to retain and use information from previous inputs.

As a second task, we use **motion prediction** (motivated by Lazar et al., 2009). In the motion prediction task, the network receives random renewal sequences of the two words “123…n” and “n….321.” These sequences can be interpreted as movement of an object in one dimension from left to right and back, that is sensed by a line array of sensors. Therefore, the task was initially set up to mimic the learning of motion-specific visual receptive fields (Lazar et al., 2009). Comparing the counting task and the motion prediction task highlights their difference in respect to subsequence learning of the individual words. For the motion prediction task, all subsequences (e.g., 12, 23, 34, …) of the word “123…n” can be learned independently. This is not the case in the counting task, where, for example, the input condition b_3_ (“abbb”) cannot be learned before b_2_ (“abb”) is learned.

As a third task, we used the **occluder** task (Lazar et al., 2009), that is a combination of the counting and motion task. With *n* = 8, the input consists of random alternations of four words: “12345678,” “87654321,” “19999998,” and “89999991.” As in the motion prediction task, they can be interpreted as the movement of an object sensed by a line of sensors. To model the occlusion of part of the sensors, two additional words “19999998” and “89999991” are used. Here, the symbol “9” represents the lacking information about the object position when the object is occluded. Note that because the first letter occurs more than once, the second letter in a word is unpredictable, and therefore excluded from the performance measure.

As a fourth task, we measure the **memory capacity** (corresponds to the RAND task in Toutounji and Pipa, 2014). Here, the network receives a random sequence of symbols and has to reproduce the symbol from *n* steps back. The number of symbols used here is 6.

As a fifth task, we use the **Markov-85** task (Toutounji and Pipa, 2014). For this task, we generate an input sequence that consists of symbols generated by a first order Markov chain. The chain has six states: 1, 2, 3, 4, 5, 6. The transition probabilities for 1→2, 2→3, 3→4, 4→5, 5→6, and 6→1 are *p* = 0.85. All other transition probabilities are *p* = 0.03. The goal of the task is either to recall the inputs from *n* steps back or to predict the state *n* steps in the future.

As a sixth task, we use the **parity** task (Toutounji and Pipa, 2014). Here, the network receives a series of binary values and has to compute the parity for the current input and *n* - 1 previous inputs. This task tests the capability of the network for non-linear computation.

As a seventh task, we designed a sequence or **motion generation** task, where the network has to generate either “123…n” or “n….321.” This is the only task, where the network receives no input. The number of symbols, and thus input dimensions, is *n*, and the longer the sequence, the harder the task. Similar to the motion prediction task, this task can be interpreted as the movement of an object in one dimension. More generally, success in this task shows that the network can generate an arbitrary symbol sequence with the same symbol distribution as in the motion words. The reinforcement of two words instead of one is more difficult, because the same output symbol can be rewarded for two different reasons.

The last task is a **pattern recognition** task, designed to highlight the effect of reward-modulated changes of synaptic weights in the recurrent network. Here, the network receives random alternations of the four words “1234,” “4321,” “4213,” “2431” and has to recognize the word “1234”: the output for every letter in this word has to be 1, and for all others, 0. In this task only one output neuron was used.

## Results

**Figure 2:**
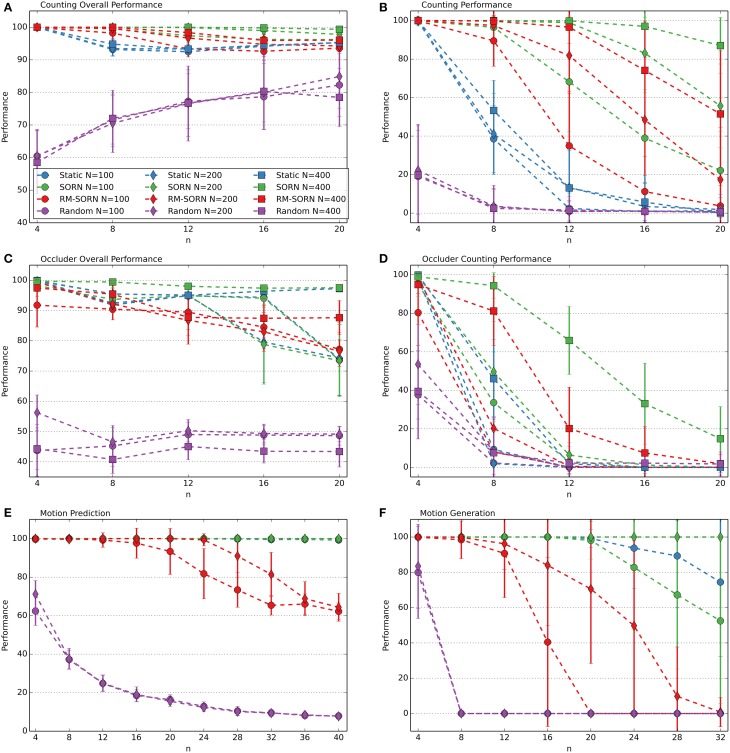
**Performance comparison in the counting (A,B), occluder (C,D), motion prediction (E), and motion generation task (F)**. Varied are the task difficulty *n* and the network size *N*. Shown is the average performance in percent over 10 data sets with 10 networks per data set. In case of motion generation, which has no input, the average performance of 100 networks is shown. Error bars indicate standard deviation.

**Figure 3:**
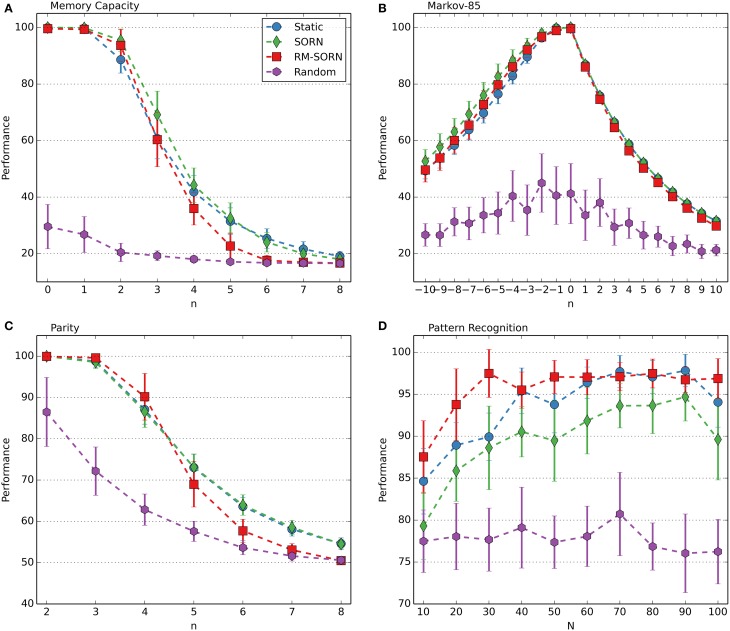
**Performance comparison in the memory capacity (A), Markov-85 (B), parity (C), and pattern recognition task (D)**. In the pattern recognition task, the network size *N* was varied. In the other tasks, the task difficulty *n* was varied with *N* = 100. Shown is the average performance over 10 data sets with 10 networks per data set. Error bars indicate standard deviation.

The performance measures for the counting, occluder, motion prediction and motion generation tasks are shown in Figure 2. The results for the Markov-85, memory capacity, parity, and pattern recognition tasks are shown in Figure 3. In most tasks, RM-SORN achieves high accuracy, and is only slightly worse than SORN. This is remarkable, considering that RM-SORN learns in a self-organized manner through interaction of plasticity mechanisms, while SORN learns through a supervised, mathematically derived algorithm.

Reward-modulation of the recurrent layer improved performance only in the pattern recognition task: it allowed RM-SORN to outperform SORN for small network sizes. In the other tasks, reward-modulation of the recurrent layer didn't improve performance and was not applied during the experiments.

### Prediction, recall and non-linear computation

In the counting task, RM-SORN achieves a high overall performance (Figure 2A) in the range 95–100% and has a counting performance (Figure 2B) in-between static and SORN networks. Higher *n* increases the difficulty for the prediction of the last letter, as the network needs to remember more of the past inputs, but reduces the overall difficulty of predicting the other letters, which are either b's or d's. A system that just produces “b” when it sees “a” or “b” and “d,” when it sees “e” or “d” can achieve a high accuracy without being able to count—this happens in the random networks, which achieve, for example, 85% accuracy for *n* = 20. The difference between static and SORN networks is due to improvement of the representational capability of the recurrent layer through unmodulated plasticity.

The performance of RM-SORN in the occluder task (Figures 2C,D) is worse, due to the higher number of input conditions, but the task can still be solved with high accuracy for *n* ≤ 8 and good overall accuracy for *n* > 8.

In the motion prediction task (Figure 2E), high accuracy can be achieved until very high *n*—the network can learn many different input condition mappings. Random networks achieve a performance slightly above the chance level of 1/*n* for higher *n*.

Remarkable is the high accuracy of RM-SORN in the motion generation task (Figure 2F), where the network never receives any input. These results will be discussed in more detail in the next section.

The performance in the memory capacity task (Figure 3A) is similar for static, SORN and RM-SORN networks: high for low *n* and low for higher *n*, hitting the chance level of 1/6 in the end. Since the input is random, learning of effective representations through STDP in the recurrent layer is not possible, and static and SORN networks have similarly low network memory. RM-SORN stays slightly behind static and SORN networks for higher *n*, which was also observed in the other tasks.

The Markov-85 task (Figure 3B) offers a different picture—static, SORN and RM-SORN networks feature high performance, also for high negative *n* (recall—corresponds to positive *n* in the memory capacity task). The structure in the input allows an efficient representation in the recurrent layer and also a more efficient mapping of the representations in the output layer. The performance for prediction (positive *n*) is lower, since the maximal achievable performance declines exponentially with each step, because of the stochastic nature of the input sequences.

The parity task (Figure 3C) offers a picture similar to the memory capacity task, since its input is also unstructured. Nevertheless, the high performance of RM-SORN for small *n* demonstrates the capability of the model for non-linear computation.

In conclusion, RM-SORN achieves high performance in all tasks and is in most tasks in-between SORN and static networks. For complex tasks (high n), the performance deteriorates more than in SORN. A more detailed analysis of the motion generation and the pattern recognition task (Figure 3D) follows in the next sections.

### Pattern recognition

In this task, RM-SORN achieved a higher performance than SORN, especially for small network sizes. The results are shown in Figure 3D. The best average performance of 97.48% was achieved with a network with only 30 excitatory neurons. Static and SORN networks of this size stay below the 90%-mark.

The reason for the better performance of RM-SORN is the reward-modulation of the recurrent layer. In order to recognize the target word, only the representations of parts of the target word are necessary, and all other symbol combinations can be ignored. SORN tries to create representations of all occurring symbol combinations and has therefore less memory for each individual combination. This leads to a poor performance with small networks. RM-SORN, on the other hand, is only rewarded if it recognizes the target word, and its recurrent layer is plastic only during this time—it learns to represent parts of the target word exclusively.

**Figure 4:**
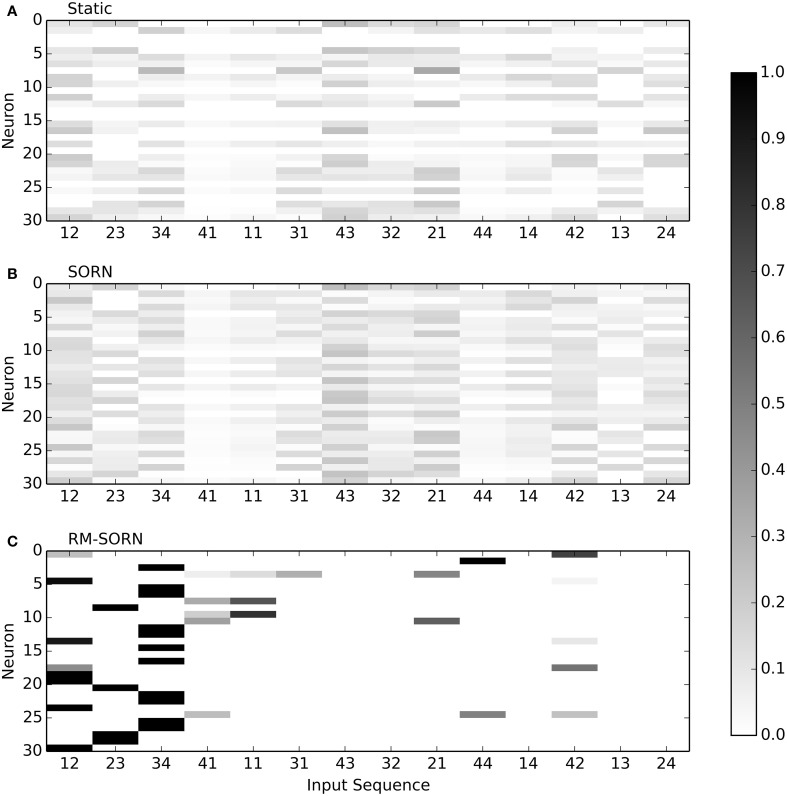
**Neuron selectivity in the recurrent layer for the pattern recognition task with 30 neurons**. Shown is the probability of a neuron to spike for each possible two-symbol input sequence. For example, the 30th neuron in RM-SORN **(C)** is activated only by the sequence “12.” Static **(A)** and SORN **(B)** are less selective.

Figure 4 visualizes the selectivity of neurons in the recurrent layer for static, SORN and RM-SORN networks with 30 neurons. For all two-symbol input sequences that occurred during testing, the probability of a neuron to spike was estimated by counting the occurred spikes. In static and SORN networks, neurons have no preferred input stimuli. The selectivity for partial sequences “43” and “21” in SORN is slightly higher than in static networks, because both occur in two patterns, while the other combinations occur only in one. In RM-SORN, neurons mostly encode the parts of the target word (“12,” “23,” “34”), which allows for a simple and effective mapping to the output neuron and a high performance.

The pattern recognition task is also the only task, where static networks perform better then SORN networks. This is due to the training procedure, as explained in section Model Evaluation: in the pattern recognition task, static networks were created by shuffling the SORN weights and taking the best out of 200 networks (in other tasks, out of 20 because of the computational load). While all intermediary evaluated SORN networks try to map all input conditions equally, some static networks, by chance, better represent the target word parts. Thus, the best static network can achieve a better performance then the best SORN network.

### Motion generation

The performance is computed as the percentage of symbols that belong to a target word. Despite rewarding both words, for *n* > 4 the network learns to generate only one word. The performance results are shown in Figure 2F. The performance of RM-SORN is impressive, since, in contrast to SORN, which learns with teacher-forcing (Jaeger, 2001), RM-SORN does not receive any external teaching signal, except the reward, and is still capable of generating the desired behavior.

**Figure 5:**
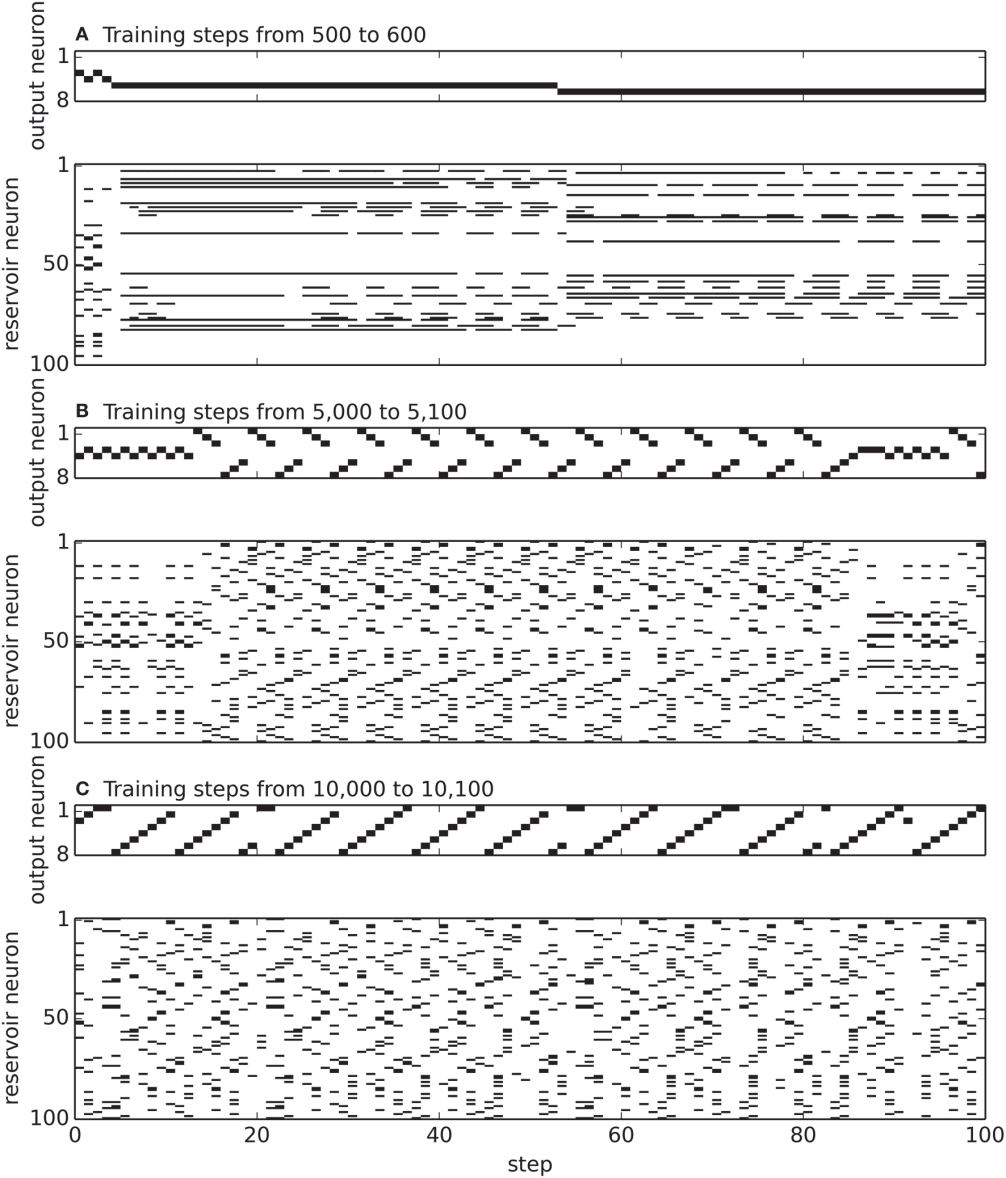
**Neural activity during reward-modulated learning in the motion generation task with *n* = 8 and *N* = 100**. The narrow tile visualizes the spiking activity of the eight output neurons and the wide tile the activity of the 100 neurons in the recurrent layer. Neuron *i* allocates the *i*-th row of the tile. In the output layer the *i*-th neuron represents the *i*-th output symbol. A diagonal in the output tile corresponds therefore either to “1234568” or “87654321.” **(A)** shows the activity from steps 500 to 600, **(B)** from 5000 to 5100 and **(C)** from 10,000 to 10,100.

Figure 5 visualizes the activity in the recurrent and output layer during 100 steps of reward-modulated plasticity at different time points during training. In the beginning, the recurrent and output activity is almost constant and has no resemblance to the target words “12345678” and “87654321.” Then, reward-modulated STDP adapts the output weights, and through feedback, changes the dynamics in the recurrent layer. As can be seen from the output activity in Figure 4B, the network generates alternately “123” and “876”—the beginnings of the target words. After an additional 5000 steps of reward-modulation, the network settles on the generation of “87654321”.

### Exploration during motion generation

**Figure 6:**
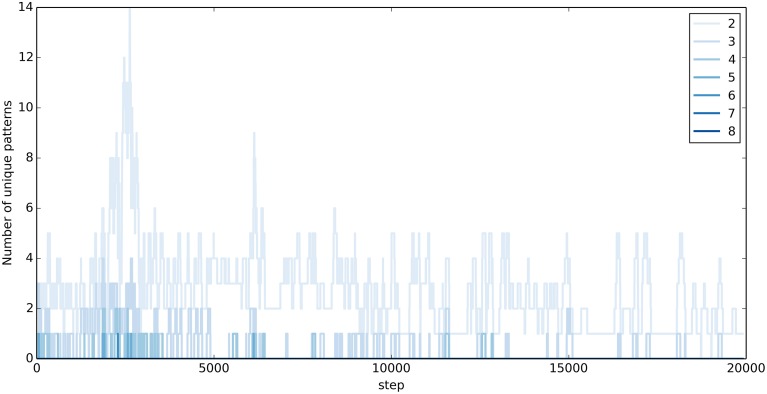
**Exploration during training in the Motion Generation Task**. Shown is the number of unique output sequences produced during the previous 100 steps, which are not part of the target words. The stronger the color, the longer the unique sequence.

In the motion generation task, exploration in the output layer corresponds to the production of different output sequences. Figure 6 visualizes the extent of exploration during 20,000 steps of training. Shown is the number of unique output sequences of different lengths in the previous 100 steps, which don't contain any parts of the target words. Exploration is highest during the first 10,000 steps. With time, the output sequences resemble more and more the target words and only few, short, original output sequences are produced. Notably, the exploration doesn't stop completely.

### Reward-modulation in the recurrent layer

The pattern recognition task was the only task in which the reward-modulation of the recurrent layer improved the performance. Punishment in the recurrent layer prevented learning of unnecessary input conditions and allowed neurons selective for the relevant input conditions to emerge. However, the pattern recognition task is the only task, where only a part of the input sequences is relevant. In the other tasks, all input conditions matter. Reward-modulation of the recurrent layer (either suppression or inversion of STDP for wrong outputs) worsened the performance. Enhancement of the exploration of the recurrent layer state space through reward-modulation of the recurrent layer was not observed. The effect of reward-modulation in the recurrent layer on the performance is visualized in the supplementary material.

### Effect of synaptic normalization of the weights to the output layer

**Figure 7:**
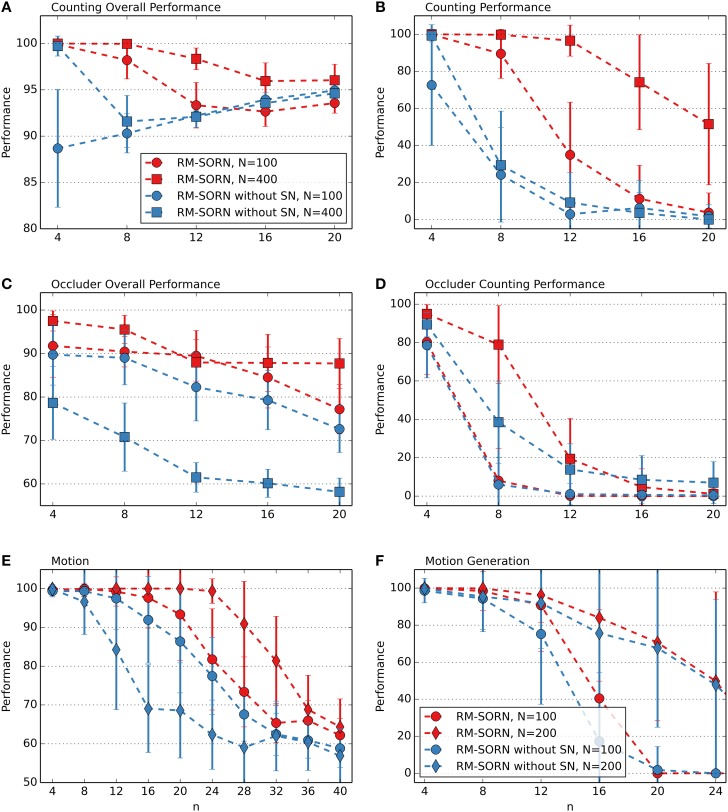
**Comparison of RM-SORN with and without SN in the counting (A,B), occluder (C,D), motion (E), and motion generation task (F)**. Shown is the average performance for different task complexity values *n*. In the motion generation task, the results are averaged over 100 networks, in the other tasks the results are averaged over 10 data sets with 10 networks per data set. Error bars indicate standard deviation.

In the recurrent layer, SN is applied to the weights between the neurons, decorrelating them and preventing seizure-like activity (Lazar et al., 2009). In the output layer, SN is applied to the weights from the recurrent to the output neurons. Since in the output layer the neurons are not interconnected and only one neuron is activated at a time (WTA), correlated activity is not possible. The performance comparison of RM-SORN with and without SN, as shown in Figure 7, suggests another effect. In the figure, only the performance results for the counting, occluder, motion prediction and motion generation task are shown—in the other tasks the performance differences are negligible.

Depression of the weights from the recurrent to the output layer happens either through punishment—Anti-STDP or SN. The challenge in the counting task is to map the representations of long, similar sequences to the corresponding output neurons. When, through chance, such a representation is mapped correctly, STDP reinforces the weights from the active neurons in the recurrent layer to the activated output neuron. Then, SN scales the weights hereby reducing the weights from the inactive recurrent neurons to the active output neuron. Thus, SN introduces synaptic competition, that leads to a stronger mapping of the representations of the long sequences.

In the motion tasks the focus is not on long sequences but on a high number of symbols. With increasing *n*, the number of symbols increases and the number of neurons representing an input decreases. Thus, less neurons represent an input condition and more neurons not related to the input condition need to be ignored and their outgoing weights depressed in order to map an input condition correctly.

The occluder task is a combination of the counting and motion task. Notably, without SN, a higher number of neurons worsens the occluder overall and the motion task performance. When there are more neurons in the recurrent layer, the output neurons have more incoming weights and can be activated more easily by the wrong input representations. In the other tasks the length of sequences, the number of mappings and the number of neurons is moderate—synaptic competition through SN does not lead to an advantage.

### Intrinsic plasticity vs. noise for exploration

Exploration of possible output mappings or output sequences is an essential part of reward-modulated learning. Most reward-modulated models use noise for exploration. Noise, however, is per definition transient and random—the rewarded behavior is not guaranteed to appear again, even with the same input and the same neuronal state. A correct model guess induced by noise might even be derogatory. For example, in a prediction task, if the correct target neuron is activated purely by chance, while the input representation in the recurrent layer is “bad” (non-distinctive for the input condition), through STDP, connections with the “wrong” neurons (neurons which don't represent input conditions or represent other input conditions) are reinforced. IP, on the other hand, is deterministic—the rewarded behavior is reproducible, and when the output is correct, it is always due to the neuronal structure and not to chance. It is therefore not surprising that the performance results confirm the superiority of IP.

The performance comparison with noise was made by disabling IP in the output layer and introducing bit-flip-noise instead: at each step, with a given probability, the active output neuron was set to zero and another randomly chosen output neuron set to one. In order to compare IP and noise in their roles as exploration drives, they have to be aligned, regarding the target average firing rate. Ensuring the target firing rate with noise is not possible, but the thresholds of the output neurons can be selected to match on average the thresholds found through IP. Therefore, before the actual task, for each network, a preliminary run with 20,000 steps with unmodulated plasticity was made and the output thresholds averaged over the values of the last 1000 steps. Then, the network was reset to its initial state, but with the threshold averages as the new threshold values.

**Figure 8:**
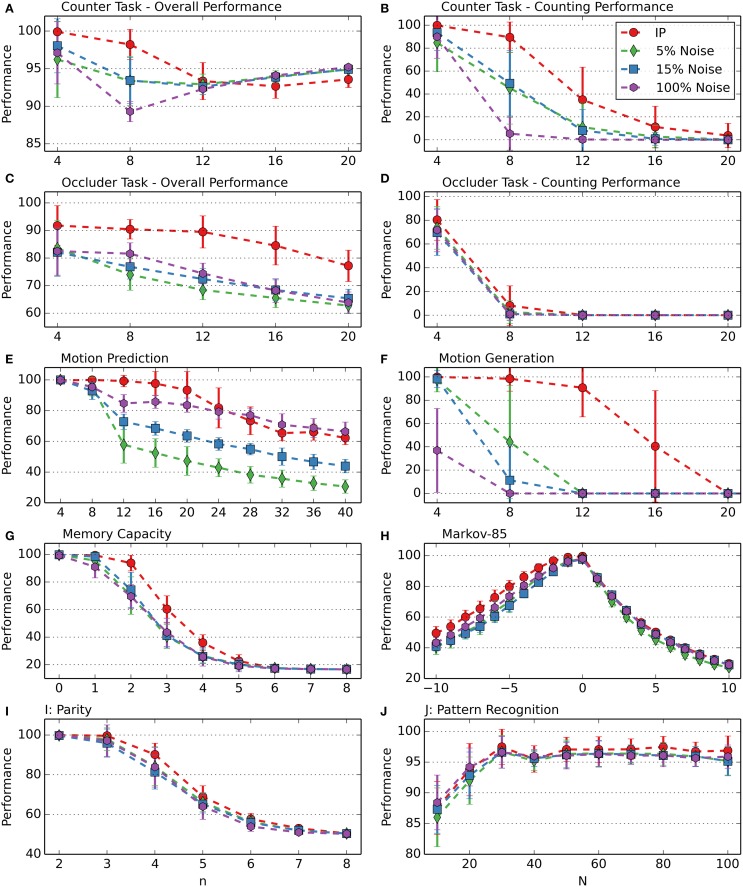
**Comparison of RM-SORN with IP and without IP but with noise at different noise levels in the counting (A,B), occluder (C,D), motion prediction (E), motion generation (F), memory capacity (G), Markov-85 (H), parity (I), and pattern recognition (J) task**. The noise probability is the probability of a randomly chosen output neuron to be activated at each training step. In the pattern recognition task, the network size *N* was varied. In the other tasks, the task difficulty *n* was varied with *N* = 100. Shown are the averages over 10 data sets with 10 networks per data set. Error bars indicate standard deviation.

During simulations without IP at different noise levels, the highest performance results were obtained with noise probabilities of 5, 15, and 100%. Figure 8 compares the performance of RM-SORN with IP and RM-SORN without IP but with noise at these levels. Overall, networks with IP achieve a higher performance than networks without IP, but with noise. Particularly motion generation would not be possible with noise as exploration drive. Noise achieves slightly higher performance in the motion task for *n* > 24, and roughly similar performance in the parity and pattern recognition task, and also in the prediction part of the Markov-85 task.

These results demonstrate the power of IP as exploration drive. Noise is a comparatively weak alternative.

## Discussion

In this article, we introduced the RM-SORN, in which reward-modulated STDP replaced supervised learning in the readout of SORN and additionally, when applied to the recurrent layer in the pattern recognition task, fitted the representation of the inputs to the task goals. RM-SORN achieved high performance comparable to that of supervised trained networks in all tasks. For complex tasks (high *n*), the performance deteriorated more strongly than in SORN, from which we can conclude that in RM-SORN, similar representations in the recurrent layer cannot be differentiated as well as in SORN. This is not surprising, considering that supervised methods have an exact error signal, while reward-modulated learning has only a general goodness signal and works on a trial and error basis.

### Reinforcement learning and reward-modulation

RM-SORN and similar models are a form of reinforcement learning, where the state is defined by the network activation and the action by the readout. The weights are adapted to maximize the reward. In a recent review of reinforcement learning in cortical networks, Senn and Pfister (2014) generalize the weight update rule to follow (10), with R being the reward and PI a plasticity induction based on pre- and postsynaptic activity. The hypothesis, that synaptic plasticity is driven by the covariance between reward and neural activity was initially introduced by Loewenstein and Seung (2006).

(10) △w=R ∗ PI

Senn and Pfister differentiate between policy gradient methods, where the average policy induction <PI> = 0 and Temporal Difference (TD) methods, where the average reward <R> = 0. The postulated purpose of these restrictions is to prevent systematic weight drift. A simple alternative to <R> = 0 is to subtract the average reward from the modulating factor as is done in RM-SORN with the rewarding strategy Mk. A similar method was used by Hoerzer et al. (2014).

TD learning with SORN was implemented by Franz (2010): the readout was replaced with action neurons and the weights modulated via the TD error. The network was able to learn symbolical sequences. A more complex actor-critic network was implemented by Frémaux et al. (2013) based on simplified spike response model neurons and used to solve a version of the cartpole task. Most recently, Dasgupta et al. (2014) developed a model, consisting of a recurrent neural network critic model representing the basal ganglia and a feed-forward correlation-based learning model representing the cerebellum. This combinatorial model was validated by letting it control a robot to forage in an enclosed environment. These increasingly complex, biologically motivated models of reward-based reinforcement learning in neural networks are able to solve complex tasks, but neglect other forms of synaptic plasticity.

### Reward-modulated STDP models and exploration

The core of RM-SORN is the interaction of IP and STDP: IP explores possible output mappings or output sequences, and STDP reinforces the rewarded ones. In contrast to most previous models, noise for exploration is neither necessary nor desirable, as IP is considerably superior to noise in most tasks and on the same level in the rest.

Previous reward-modulated models, with one exception, use only STDP or a hebbian rule, and noise for exploration. Legenstein et al. (2008) performed an extensive analytical and simulational analysis of reward-modulated STDP. Their networks made from noisy, leaky integrate-and-fire neurons solved several tasks: increasing the firing rate of a single neuron, learning of spike times, spike pattern discrimination and isolated digit recognition. One of their findings was that spontaneous activity is essential for reward-modulated learning in order to explore which firing patterns are rewarded.

Shortly before Legenstein, in 2007, Izhikevich created a model with spiking neurons, where the distal reward problem was solved through eligibility traces and reward-modulated STDP (Izhikevich, 2007). He validated his model on three simulations: reinforcement of a synapse between two excitatory neurons, classical (Pavlovian) conditioning, and stimulus-response-instrumental conditioning. During learning, spontaneous activity was achieved through random input, mimicking noisy miniature PSPs. Izhikevich concluded that STDP is insensitive to random firings during the waiting time for the reward, and is only triggered by precise firing patterns in the millisecond range, which are rare. He also argued that the precise timing of spikes is essential for reinforcement with STDP, and that this effect could not be reproduced with firing rate models. This statement was disproved by Soltoggio and Steil (2013).

Soltoggio and Steil reproduced most of the experiments of Izhikevich in a rate-based model, and they showed that classical and instrumental conditioning with delayed rewards can be learned without precise spike timing. Their Rare Correlation Model features a rate-based hebbian rule with a threshold that allows only the upper 1% of all correlations to be applied. Noise is added to the firing rate after the tanh-activation, to generate spontaneous activity. Beside the tasks from Izhikevich, the model was also successfully applied in robotics for classical and operant conditioning of the humanoid robot iCub (Soltoggio et al., 2013).

Another rate-based model with reward-modulated hebbian learning was created by Hoerzer et al. (2014). It consists of a recurrent layer and a linear readout with a feedback connection to the recurrent layer. In Hörzer's model, the recurrent layer is chaotic with tanh-neurons, following Sussillo and Abbott (2009), but instead of supervised learning, a reward-modulated hebbian rule is used to train the readout. The model was successfully applied to several tasks, including periodic pattern generation and non-linear analog computations on complex input signals. During learning, noise was applied to the firing rates of neurons in the readout. The authors point out that this exploration noise is the driving force for learning, and that without it, no learning would take place.

Other notable reward-modulated spiking neuron models include Soltani and Wang (2010), where a connection between reward-modulated plasticity and probabilistic inference was observed and Bourjaily and Miller (2011), where reward-modulated STDP combined with multiplicative synaptic scaling was used to learn a XOR task.

The most recent model combines reward-modulated STDP with eligibility traces, IP and synaptic scaling in a 2-layer network with binary thresholded units (Savin and Triesch, 2014), similar to the model in this article. The model was applied to two working memory tasks: delayed response and delayed categorization. During learning, task-dependent representations beneficial for task performance emerged in the recurrent layer. The neurons were noisy, but the noise did not play any special role in learning.

The focus in these publications is on the hebbian or STDP learning rule and tasks with delayed reward. The interaction of several plasticity mechanisms and the role, that homeostatic plasticity plays in reward-modulated learning was not investigated. This article demonstrates that reward-modulated learning can achieve performance comparable to that of supervised learning methods in tasks of different nature and complexity, and that IP can serve as the exploratory drive during learning. This is at first glance surprising, since IP is a homeostatic mechanism. However, IP as implemented here ensures an average firing rate by lowering or raising the thresholds continuously and these threshold-changes alter the neuronal activity and drive the exploration.

### Outlook

Supervised learning requires a precise error signal, which is probably not present in the brain. For reward-modulated learning, only a general goodness signal is necessary. Additionally, it can be applied to any network structure. In this article, only two-layer networks were investigated, which in itself is biologically not plausible. More complex network architectures, which present a challenge for supervised training methods, may, in contrast, unfold the capabilities of reward-modulated learning.

Another possible line of investigation is the effect and nature of modulation via the reward-prediction error. In all tasks, except the counting- and pattern prediction-task, modulation via the reward-prediction error was better than modulation via the reward directly. The difference in the task performances offers a starting point for a more detailed investigation.

An interesting question is also, how exploration and exploitation can be balanced. During reward-modulated plasticity the exploration diminishes, but never stops completely, which made it necessary to evaluate intermediate networks in order to get the best performance (as described in sections Model Evaluation and Exploration during Motion Generation). From a functional point of view, a mechanism that stops exploration, when a sufficient performance level is achieved, is desirable. The rewarding strategy seems to be a good place for such a mechanism.

### Conflict of interest statement

The authors declare that the research was conducted in the absence of any commercial or financial relationships that could be construed as a potential conflict of interest.

## Supplementary material

The Supplementary Material for this article can be found on line at: http://www.frontiersin.org/journal/10.3389/fncom.2015.00036/abstract

Effect of reward-modulation of the recurrent layer on performance, 603 parameters for the tasks, detailed performance results (means and standard deviation).
