# Supplementary material for: RM-SORN: a reward-modulated self-organizing recurrent neural network
Source: Front Comput Neurosci. 2015 Mar 24;9:36. doi: 10.3389/fncom.2015.00036 (PMC4371712; doi:10.3389/fncom.2015.00036)
Supplement: Supplementary file 1 [file DataSheet1.PDF]

# *Supplementary Material*

# RM-SORN: A Reward-Modulated Self-Organizing Recurrent Neural Network

**Witali Aswolinskiy*, Gordon Pipa**

Institute of Cognitive Science, University of Osnabrück, Osnabrück, Lower Sasony, Germany

***Correspondence: Witali Aswolinskiy, waswolinskiy@uos.de**

## 1. Reward-Modulation in the Recurrent Layer

The difference between an unmodulated plastic network and RM-SORN is the suppression or inversion of STDP. The inversion of STDP – Anti-STDP can be viewed as punishment. In the output layer, punishment deletes wrong mapping. In the recurrent layer, punishment prevents learning of unnecessary input conditions, as was demonstrated in the pattern recognition task in section 4.4. However, the pattern recognition task is the only task, where only a part of the input sequences is relevant. In all other tasks, as seen in the figure below, reward-modulation of the recurrent layer doesn't improve performance.

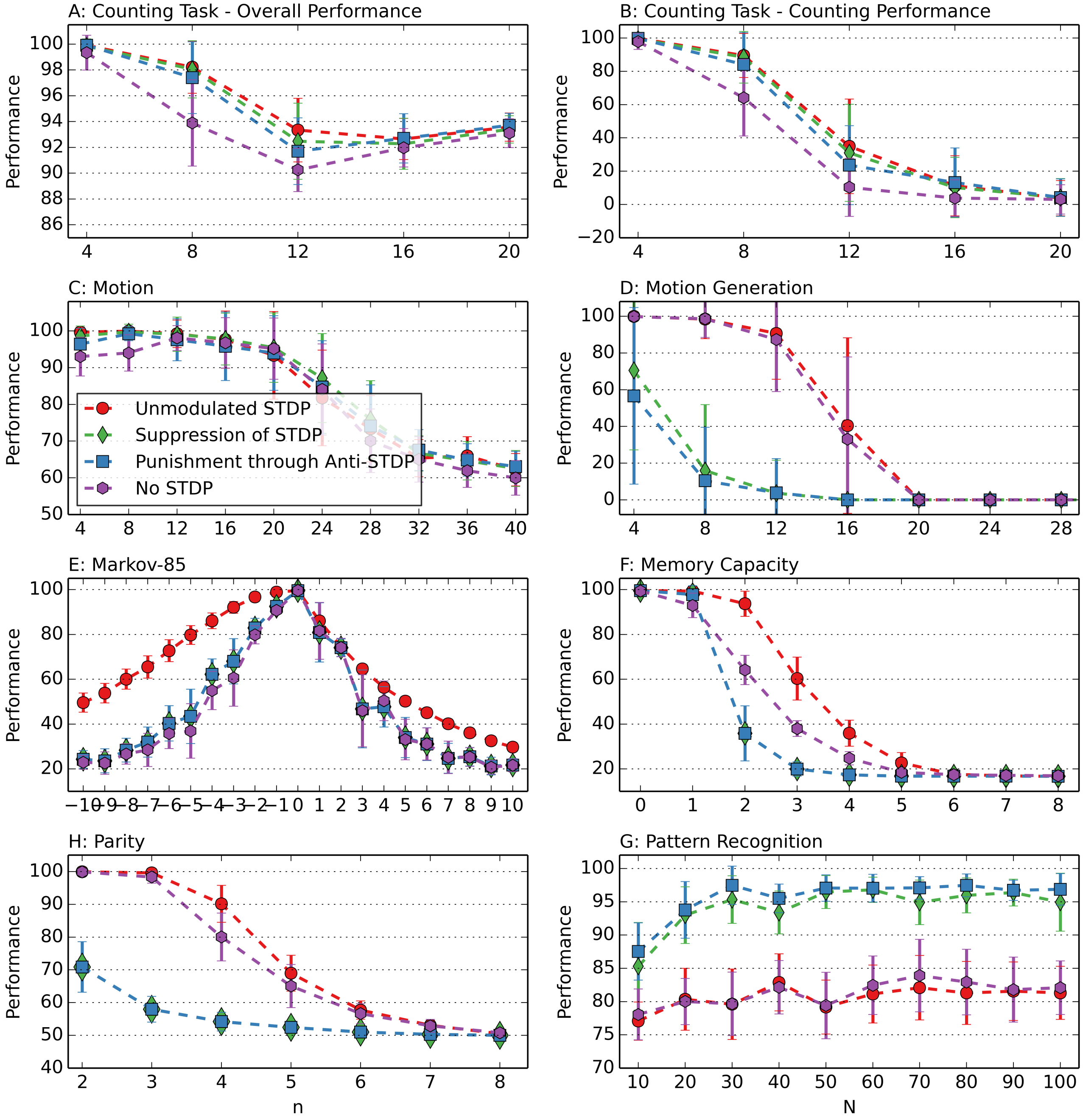


**Figure 1:** Comparison of different modulation types in the recurrent layer. Unmodulated STDP: The recurrent layer is not modulated – STDP is applied at each step. Suppression of STDP: For wrong outputs STDP is suppressed. Punishment through Anti-STDP: For wrong outputs STDP is inversed to Anti-STDP. No STDP: The weights in the recurrent layer are static. x-axis: task complexity n or, in case of pattern recognition (F) number of neurons N. Shown are the average performances over ten data sets with ten networks per data set. Error bars indicate standard deviation.

## 2. Parameters for the Tasks

In this section, the model parameters for selected task configurations are listed. The choices for the model include:

- The recurrent connectivity applies to the average: For example, with a recurrent connectivity of 10% and 100 neurons, a neuron receives input on average from 10 other neurons, but there will be neurons with more and less connections.
- The input connectivity is given per input. For example, with an input connectivity of 10% and N=100, each input symbol projects to exactly 10 different neurons.
- Non-overlapping input neurons: A neuron receives input only form one input channel (one symbol). If the current input is this symbol, the neuron receives '1' as input.
- The weights are initially drawn from a normal distribution

**Counting Task**
No modulation of the recurrent layer; punishment; rewarding strategy M0

**Supplementary Table 1: Network parameters for the counter task with N=100**

| | | | | | | | |
|---|---|---|---|---|---|---|---|
| Recurrent Layer | | | | | | | |
| $N^E$ | 100 | Recurrent Connectivity | 5% | $\eta_{STDP}$ | 0.001 | $\eta_{IP}$ | 0.002 |
| $\mu_{IP}$ | 0.15 | Input Connectivity | 5% | $T_E$ max | 1 | $T_I$ max | 1 |
| Output Layer | | | | | | | |
| $N^E$ | n | $\mu_{IP}$ | a,c,e,f: 0.05<br>b,d: 0.4 | $T_E$ max | 0.5 | $\eta_{STDP}$ | 0.005 | $\eta_{IP}$ | 0.005 |

**Supplementary Table 2: Network parameters for the counter task with N=200**

| | | | | | | | |
|---|---|---|---|---|---|---|---|
| Recurrent Layer | | | | | | | |
| $N^E$ | 200 | Recurrent Connectivity | 5% | $\eta_{STDP}$ | 0.005 | $\eta_{IP}$ | 0.001 |
| $\mu_{IP}$ | 0.1 | Input Connectivity | 10% | $T_E$ max | 1 | $T_I$ max | 1 |
| Output Layer | | | | | | | |
| $N^E$ | n | $\mu_{IP}$ | a,c,e,f: 0.05<br>b,d: 0.4 | $T_E$ max | 0.5 | $\eta_{STDP}$ | 0.001 | $\eta_{IP}$ | 0.005 |

**Supplementary Table 3: Network parameters for the counter task with N=400**

| | | | | | | | | | |
|---|---|---|---|---|---|---|---|---|---|
| Recurrent Layer | | | | | | | | | |
| $N^E$ | 400 | Recurrent Connectivity | | 1.25% | $\eta_{STDP}$ | 0.005 | $\eta_{IP}$ | 0.002 | |
| $\mu_{IP}$ | 0.1 | Input Connectivity | | 10% | $T_E$ max | 1 | $T_I$ max | 1 | |
| Output Layer | | | | | | | | | |
| $N^E$ | n | $\mu_{IP}$ | a,c,e,f: 0.05<br>b,d: 0.4 | $T_E$ max | 0.5 | $\eta_{STDP}$ | 0.001 | $\eta_{IP}$ | 0.001 |

## Motion Task

No reward-modulation of the recurrent layer; punishment; rewarding strategy M20

**Supplementary Table 4: Network parameters for the motion task with N=200**

| | | | | | | | | | |
|---|---|---|---|---|---|---|---|---|---|
| Recurrent Layer | | | | | | | | | |
| $N^E$ | 200 | Recurrent Connectivity | | 5% | $\eta_{STDP}$ | 0.001 | $\eta_{IP}$ | 0.002 | |
| $\mu_{IP}$ | 0.2 | Input Connectivity | | 15% | $T_E$ max | 1 | $T_I$ max | 1 | |
| Output Layer | | | | | | | | | |
| $N^E$ | n | $\mu_{IP}$ | 1/n | $T_E$ max | 1 | $\eta_{STDP}$ | 0.005 | $\eta_{IP}$ | 0.005 |

## Occluder Task

No reward-modulation of the recurrent layer; punishment; rewarding strategy M10

**Supplementary Table 5: Network parameters for the occluder task with N=400**

| | | | | | | | | | |
|---|---|---|---|---|---|---|---|---|---|
| Recurrent Layer | | | | | | | | | |
| $N^E$ | 400 | Recurrent Connectivity | | 1% | $\eta_{STDP}$ | 0.005 | $\eta_{IP}$ | 0.001 | |
| $\mu_{IP}$ | 0.1 | Input Connectivity | | 5% | $T_E$ max | 1 | $T_I$ max | 1 | |
| Output Layer | | | | | | | | | |
| $N^E$ | n | $\mu_{IP}$ | $\mu_{Occl.}$ | $T_E$ max | 1 | $\eta_{STDP}$ | 0.001 | $\eta_{IP}$ | 0.001 |

$\mu_{Occl.}$ is determined according to the following calculation

- first (and n-th) symbol appears in each word:
  first and n-th number: 4/(n*4)
- numbers between first and n appear in 2 words once:
  numbers between first and n-th: 2*(n-2)/(n*4)
- occluder appears in two words n-2 times:
  2.0*(n-2)/(n*4)

**Motion Generation Task**

No modulation of the recurrent layer; no punishment in the output layer; rewarding strategy M10

**Supplementary Table 6: Network parameters for the motion generation task with N=200**

| | | | | | | | | |
|---|---|---|---|---|---|---|---|---|
| Recurrent Layer | | | | | | | | |
| $N^E$ | 200 | Recurrent Connectivity | | 5% | $\eta_{STDP}$ | 0.01 | $\eta_{IP}$ | 0.001 |
| $\mu_{IP}$ | 0.1 | Input Connectivity | | 5% | $T_E$ max | 1 | $T_I$ max | 1 |
| Output Layer | | | | | | | | |
| $N^E$ | n | $\mu_{IP}$ | 1/n | $T_E$ max 0.5 | | $\eta_{STDP}$ | 0.01 | $\eta_{IP}$ 0.005 |

**Parity Task**

No reward-modulation of the recurrent layer; No IP in the recurrent layer With IP, the results are considerably worse; No punishment; Rewarding strategy M10.

**Supplementary Table 7: Network parameters for the parity task**

| | | | | | | | | | |
|---|---|---|---|---|---|---|---|---|---|
| Recurrent Layer | | | | | | | | | |
| $N^E$ | 100 | Recurrent Connectivity | | | 5% | $\eta_{STDP}$ | 0.001 | $\eta_{IP}$ | 0.005 |
| $\mu_{IP}$ | 0.1 | Input Connectivity | | | 40% | $T_E$ max | 1 | $T_I$ max | 1 |
| Output Layer | | | | | | | | | |
| $N^E$ | n | $\mu_{IP}$ | 0.5 | $T_E$ max | 0.5 | $\eta_{STDP}$ | 0.001 | $\eta_{IP}$ | 0.005 |

**Memory Capacity Task**

No reward-modulation of the recurrent layer; no punishment; rewarding strategy M5

**Supplementary Table 8: Network parameters for the memory capacity task**

| Recurrent Layer | | | | | | | | |
|---|---|---|---|---|---|---|---|---|
| $N^E$ | 100 | Recurrent Connectivity | | 5% | $\eta_{STDP}$ | 0.005 | $\eta_{IP}$ | 0.001 |
| $\mu_{IP}$ | 0.1 | Input Connectivity | | 10% | $T_E$ max | 1 | $T_I$ max | 1 |
| Output Layer | | | | | | | | |
| $N^E$ | n | $\mu_{IP}$ | 1/n | $T_E$ max | 0.5 | $\eta_{STDP}$ | 0.0005 | $\eta_{IP}$ | 0.0005 |

**Markov-85 Task**
No reward-modulation of the recurrent layer; no punishment; rewarding strategy M5

**Supplementary Table 9: Network parameters for the Markov-85 task**

| Recurrent Layer | | | | | | | | | |
|---|---|---|---|---|---|---|---|---|---|
| $N^E$ | 100 | Recurrent Connectivity | | | 5% | $\eta_{STDP}$ | 0.005 | $\eta_{IP}$ | 0.001 |
| $\mu_{IP}$ | 0.2 | Input Connectivity | | | 10% | $T_E$ max | 1 | $T_I$ max | 1 |
| Output Layer | | | | | | | | | |
| $N^E$ | n | $\mu_{IP}$ | 1/6 | $T_E$ max | 0.5 | $\eta_{STDP}$ | 0.005 | $\eta_{IP}$ | 0.001 |

**Pattern Recognition Task**
Reward-modulation in both layers; punishment; rewarding strategy M0

**Supplementary Table 10: Network parameters for the pattern recognition task**

| Recurrent Layer | | | | | | | | | |
|---|---|---|---|---|---|---|---|---|---|
| $N^E$ | 30 | Recurrent Connectivity | | | 30% | $\eta_{STDP}$ | 0.005 | $\eta_{IP}$ | 0.001 |
| $\mu_{IP}$ | 0.05 | Input Connectivity | | | 25% | $T_E$ max | 1 | $T_I$ max | 1 |
| Output Layer | | | | | | | | | |
| $N^E$ | n | $\mu_{IP}$ | 1/4 | $T_E$ max | 0.5 | $\eta_{STDP}$ | 0.0001 | $\eta_{IP}$ | 0.001 |

## 3. Peformance Results

Following, for each experiment, mean performance and the standard deviation over 100 trials in percent are listed.

### Counting Task

**Supplementary Table 11: Counting Task - Overall Performance, N=100**

| n | 4 | 8 | 12 | 16 | 20 |
|---|---|---|---|---|---|
| **Static** | 99.94 (0.51) | 93.17 (1.93) | 92.49 (0.62) | 94.18 (0.34) | 95.30 (0.33) |
| **SORN** | 99.98 (0.06) | 99.54 (1.07) | 97.40 (2.17) | 96.25 (1.39) | 96.21 (0.95) |
| **RM-SORN** | 99.91 (0.37) | 98.21 (2.02) | 93.34 (2.46) | 92.65 (1.60) | 93.57 (1.09) |
| **Random** | 60.45 (8.25) | 71.55 (7.40) | 77.28 (8.41) | 78.66 (10.13) | 82.27 (9.76) |

**Supplementary Table 12: Counting Task - Counting Performance; N=100**

| n | 4 | 8 | 12 | 16 | 20 |
|---|---|---|---|---|---|
| **Static** | 99.74 (2.54) | 38.57 (17.42) | 2.39 (8.06) | 0.89 (5.76) | 1.36 (6.67) |
| **SORN** | 99.94 (0.24) | 96.48 (8.61) | 68.17 (25.93) | 38.97 (23.51) | 22.17 (20.26) |
| **RM-SORN** | 99.87 (0.49) | 89.49 (13.23) | 34.92 (28.44) | 11.20 (18.07) | 3.73 (10.59) |
| **Random** | 19.02 (24.03) | 3.29 (9.56) | 0.88 (4.92) | 1.04 (4.94) | 0.41 (2.65) |

**Supplementary Table 13: Counting Task - Overall Performance, N=200**

| n | 4 | 8 | 12 | 16 | 20 |
|---|---|---|---|---|---|
| **Static** | 100.00 (0.01) | 93.44 (2.32) | 93.33 (1.52) | 94.33 (0.71) | 95.33 (0.38) |
| **SORN** | 99.99 (0.01) | 100.00 (0.00) | 99.91 (0.27) | 98.96 (1.12) | 97.81 (1.16) |
| **RM-SORN** | 100.00 (0.01) | 99.61 (1.01) | 96.68 (1.91) | 94.75 (2.37) | 94.26 (1.48) |
| **Random** | 60.52 (7.77) | 70.51 (8.97) | 76.59 (11.52) | 80.09 (11.43) | 84.88 (9.70) |

**Supplementary Table 14: Counting Task - Counting Performance; N=200**

| n | 4 | 8 | 12 | 16 | 20 |
|---|---|---|---|---|---|
| **Static** | 99.99 (0.03) | 41.11 (21.01) | 13.37 (19.85) | 3.46 (12.12) | 1.90 (7.90) |
| **SORN** | 99.98 (0.04) | 100.00 (0.00) | 98.88 (3.45) | 83.06 (18.76) | 55.68 (21.79) |
| **RM-SORN** | 100.00 (0.01) | 97.46 (8.32) | 81.82 (19.74) | 48.43 (32.59) | 17.35 (27.29) |
| **Random** | 22.25 (23.04) | 3.61 (10.81) | 0.91 (5.60) | 1.16 (5.18) | 0.42 (2.72) |

**Supplementary Table 15: Counting Task - Overall Performance; N=400**

| n | 4 | 8 | 12 | 16 | 20 |
|---|---|---|---|---|---|
| **Static** | 99.99 (0.06) | 94.81 (1.74) | 93.31 (1.48) | 94.46 (0.81) | 95.24 (0.00) |
| **SORN** | 99.99 (0.01) | 99.99 (0.03) | 99.98 (0.06) | 99.79 (1.00) | 99.36 (0.70) |
| **RM-SORN** | 100.00 (0.01) | 99.97 (0.28) | 98.35 (1.17) | 95.95 (1.96) | 96.04 (1.73) |
| **Random** | 58.52 (9.67) | 72.03 (8.63) | 76.67 (10.24) | 80.32 (9.38) | 78.48 (8.94) |

**Supplementary Table 16: Counting Task - Counting Performance; N=400**

| n | 4 | 8 | 12 | 16 | 20 |
|---|---|---|---|---|---|
| **Static** | 99.96 (0.32) | 53.31 (15.63) | 13.06 (19.28) | 5.74 (13.77) | 0.00 (0.00) |
| **SORN** | 99.99 (0.03) | 99.95 (0.30) | 99.82 (0.60) | 96.99 (13.03) | 87.03 (14.62) |
| **RM-SORN** | 100.00 (0.01) | 99.75 (2.54) | 96.57 (8.37) | 74.12 (25.61) | 51.52 (32.77) |
| **Random** | 19.87 (26.24) | 2.46 (9.92) | 1.65 (7.16) | 0.83 (5.60) | 0.78 (2.50) |

**Motion Task**

**Supplementary Table 17: Motion Task Performance; N=100**

| n | 4 | 8 | 12 | 16 | 20 | 24 | 28 | 32 | 36 | 40 |
|---|---|---|---|---|---|---|---|---|---|---|
| **Static** | 100.00 (0.00) | 100.00 (0.00) | 100.00 (0.00) | 100.00 (0.00) | 99.99 (0.13) | 99.86 (0.52) | 99.80 (0.63) | 99.52 (1.11) | 99.59 (1.00) | 99.29 (1.11) |
| **SORN** | 99.99 (0.08) | 100.00 (0.01) | 99.98 (0.10) | 99.99 (0.09) | 100.00 (0.00) | 99.99 (0.02) | 99.99 (0.02) | 99.89 (0.41) | 99.73 (0.55) | 99.64 (0.60) |
| **RM-SORN** | 99.67 (1.34) | 99.95 (0.53) | 99.24 (3.76) | 97.63 (7.80) | 93.35 (11.94) | 81.76 (13.04) | 73.37 (9.03) | 65.36 (5.04) | 65.99 (5.22) | 62.20 (4.40) |
| **Random** | 62.37 (7.47) | 37.17 (4.82) | 24.81 (3.97) | 18.53 (3.20) | 16.34 (2.58) | 12.90 (2.36) | 10.44 (2.42) | 9.50 (1.76) | 8.18 (1.62) | 7.92 (1.50) |

**Supplementary Table 18: Motion Task Performance; N=200**

| n | 4 | 8 | 12 | 16 | 20 | 24 | 28 | 32 | 36 | 40 |
|---|---|---|---|---|---|---|---|---|---|---|
| **Static** | 99.98 (0.06) | 100.00 (0.00) | 100.00 (0.00) | 100.00 (0.00) | 100.00 (0.00) | 100.00 (0.00) | 100.00 (0.00) | 100.00 (0.00) | 100.00 (0.05) | 100.00 (0.00) |
| **SORN** | 99.97 (0.08) | 99.98 (0.07) | 99.99 (0.02) | 99.98 (0.06) | 99.99 (0.05) | 99.99 (0.02) | 99.98 (0.09) | 100.00 (0.01) | 99.99 (0.02) | 99.98 (0.10) |
| **RM-SORN** | 99.98 (0.21) | 99.60 (1.60) | 100.00 (0.00) | 100.00 (0.00) | 100.00 (0.00) | 99.35 (3.17) | 90.91 (10.94) | 81.38 (11.46) | 68.83 (8.83) | 64.34 (7.22) |
| **Random** | 71.16 (7.05) | 37.57 (5.48) | 24.91 (4.34) | 19.09 (3.89) | 15.54 (2.68) | 12.32 (2.27) | 10.17 (1.98) | 9.41 (1.78) | 8.47 (1.98) | 7.70 (1.57) |

**Occluder Task**

**Supplementary Table 19: Occluder Task - Overall Performance; N=100**

| n | 4 | 8 | 12 | 16 | 20 |
|---|---|---|---|---|---|
| **Static** | 99.91 (0.24) | 92.43 (1.41) | 94.88 (0.32) | 94.37 (2.56) | 73.94 (12.04) |
| **SORN** | 98.25 (2.03) | 93.69 (1.92) | 94.90 (0.37) | 94.06 (3.00) | 73.61 (11.79) |
| **RM-SORN** | 91.77 (7.22) | 90.44 (3.52) | 89.47 (5.82) | 84.50 (7.01) | 77.18 (5.67) |
| **Random** | 43.73 (8.58) | 45.27 (6.75) | 49.02 (4.12) | 48.84 (3.24) | 48.66 (3.12) |

**Supplementary Table 20: Occluder Task - Counting Performance; N=100**

| n | 4 | 8 | 12 | 16 | 20 |
|---|---|---|---|---|---|
| **Static** | 99.81 (0.77) | 9.31 (17.10) | 0.00 (0.00) | 0.00 (0.00) | 0.00 (0.00) |
| **SORN** | 95.18 (6.50) | 33.60 (26.28) | 3.01 (9.73) | 0.00 (0.00) | 0.00 (0.00) |
| **RM-SORN** | 80.33 (17.11) | 8.05 (16.68) | 0.12 (0.89) | 0.00 (0.00) | 0.00 (0.00) |
| **Random** | 37.61 (22.86) | 2.21 (7.29) | 0.25 (2.53) | 0.00 (0.00) | 0.00 (0.00) |

**Supplementary Table 21: Occluder Task - Overall Performance; N=200**

| n | 4 | 8 | 12 | 16 | 20 |
|---|---|---|---|---|---|
| **Static** | 99.42 (1.10) | 91.83 (0.62) | 94.95 (0.20) | 79.60 (13.36) | 74.30 (12.26) |
| **SORN** | 99.76 (0.33) | 95.45 (2.15) | 95.12 (0.63) | 78.78 (13.09) | 73.40 (11.87) |
| **RM-SORN** | 99.39 (1.38) | 92.16 (2.85) | 86.74 (7.84) | 82.97 (6.41) | 76.54 (3.78) |
| **Random** | 56.25 (5.85) | 46.57 (4.86) | 50.29 (3.70) | 49.37 (3.10) | 49.15 (2.54) |

**Supplementary Table 22: Occluder Task - Counting Performance; N=200**

| n | 4 | 8 | 12 | 16 | 20 |
|---|---|---|---|---|---|
| **Static** | 98.15 (3.44) | 1.91 (8.50) | 0.00 (0.00) | 0.00 (0.00) | 0.00 (0.00) |
| **SORN** | 99.15 (1.20) | 49.64 (26.05) | 6.39 (14.73) | 1.19 (7.58) | 0.00 (0.00) |
| **RM-SORN** | 98.76 (1.85) | 20.22 (26.67) | 0.53 (3.71) | 0.00 (0.00) | 0.00 (0.00) |
| **Random** | 53.49 (20.72) | 8.40 (17.65) | 0.00 (0.00) | 0.13 (1.27) | 0.00 (0.00) |

**Supplementary Table 23: Occluder Task - Overall Performance; N=400**

| n | 4 | 8 | 12 | 16 | 20 |
|---|---|---|---|---|---|
| **Static** | 99.92 (0.15) | 95.47 (1.69) | 95.02 (0.44) | 96.41 (0.11) | 97.25 (0.13) |
| **SORN** | 99.68 (0.53) | 99.42 (0.64) | 98.02 (0.95) | 97.39 (0.77) | 97.52 (0.42) |
| **RM-SORN** | 97.46 (2.69) | 95.41 (3.60) | 87.74 (5.87) | 87.43 (6.50) | 87.64 (5.65) |
| **Random** | 44.32 (6.86) | 40.78 (4.64) | 45.04 (4.37) | 43.48 (3.86) | 43.42 (5.13) |

**Supplementary Table 24: Occluder Task - Counting Performance; N=400**

| n | 4 | 8 | 12 | 16 | 20 |
|---|---|---|---|---|---|
| **Static** | 99.87 (0.35) | 46.06 (20.44) | 1.84 (8.83) | 0.00 (0.00) | 0.00 (0.00) |
| **SORN** | 98.98 (1.95) | 94.33 (6.76) | 65.89 (17.65) | 33.09 (20.99) | 14.86 (16.67) |
| **RM-SORN** | 94.88 (4.53) | 81.20 (17.83) | 20.15 (21.46) | 7.46 (13.64) | 1.86 (4.76) |
| **Random** | 39.41 (14.30) | 7.57 (11.16) | 2.20 (5.77) | 2.19 (5.24) | 1.75 (6.22) |

**Motion Generation Task**

**Supplementary Table 25: Motion Generation Performance; N=100**

| n | 4 | 8 | 12 | 16 | 20 | 24 | 28 | 32 |
|---|---|---|---|---|---|---|---|---|
| **Static** | 100.00 (0.00) | 100.00 (0.00) | 100.00 (0.00) | 100.00 (0.04) | 99.18 (4.98) | 93.70 (22.92) | 89.27 (29.42) | 74.44 (42.18) |
| **SORN** | 100.00 (0.01) | 100.00 (0.00) | 100.00 (0.01) | 99.99 (0.04) | 98.07 (10.57) | 82.78 (35.43) | 67.21 (42.02) | 52.50 (46.90) |
| **RM-SORN** | 99.88 (1.11) | 98.44 (10.66) | 90.69 (25.01) | 40.44 (47.82) | 0.00 (0.00) | 0.00 (0.00) | 0.00 (0.00) | 0.00 (0.00) |
| **Random** | 79.96 (26.11) | 0.00 (0.00) | 0.00 (0.00) | 0.00 (0.00) | 0.00 (0.00) | 0.00 (0.00) | 0.00 (0.00) | 0.00 (0.00) |

**Supplementary Table 26: Motion Generation Performance; N=200**

| n | 4 | 8 | 12 | 16 | 20 | 24 | 28 | 32 |
|---|---|---|---|---|---|---|---|---|
| **Static** | 99.97 (0.02) | 100.00 (0.02) | 100.00 (0.00) | 100.00 (0.00) | 99.99 (0.04) | 99.99 (0.04) | 99.98 (0.07) | 100.00 (0.05) |
| **SORN** | 99.99 (0.02) | 99.98 (0.04) | 100.00 (0.01) | 100.00 (0.02) | 100.00 (0.00) | 100.00 (0.02) | 99.96 (0.11) | 99.99 (0.03) |
| **RM-SORN** | 100.00 (0.01) | 99.83 (1.10) | 96.18 (14.59) | 83.98 (34.24) | 70.58 (42.22) | 49.75 (48.18) | 9.74 (27.96) | 0.84 (8.34) |
| **Random** | 83.45 (23.81) | 0.00 (0.00) | 0.00 (0.00) | 0.00 (0.00) | 0.00 (0.00) | 0.00 (0.00) | 0.00 (0.00) | 0.00 (0.00) |

**Parity Task**

**Supplementary Table 27: Parity Performance**

| n | 2 | 3 | 4 | 5 | 6 | 7 | 8 |
|---|---|---|---|---|---|---|---|
| **Static** | 99.97 (0.07) | 98.77 (1.41) | 87.08 (3.54) | 73.01 (3.25) | 63.61 (2.13) | 58.09 (1.63) | 54.60 (1.41) |
| **SORN** | 99.97 (0.05) | 98.55 (1.51) | 86.55 (3.80) | 73.14 (3.20) | 63.96 (2.47) | 58.53 (1.71) | 54.53 (1.19) |
| **RM-SORN** | 99.93 (0.19) | 99.60 (0.66) | 90.18 (5.64) | 68.97 (5.49) | 57.72 (2.79) | 53.07 (1.52) | 50.50 (0.89) |

| | | | | | | | |
|---|---|---|---|---|---|---|---|
| **Random** | 86.49 (8.36) | 72.16 (5.86) | 62.84 (3.79) | 57.58 (2.45) | 53.63 (1.71) | 51.61 (1.20) | 50.60 (0.82) |

**Memory Capacity Task**

**Supplementary Table 28: Memory Capacity Performance**

| n | 0 | 1 | 2 | 3 | 4 | 5 | 6 | 7 | 8 |
|---|---|---|---|---|---|---|---|---|---|
| **Static** | 100.00 (0.00) | 99.80 (0.34) | 88.63 (4.73) | 60.72 (7.20) | 41.79 (5.74) | 31.43 (4.72) | 25.33 (3.44) | 21.59 (2.64) | 19.04 (1.74) |
| **SORN** | 100.00 (0.00) | 99.87 (0.14) | 95.48 (4.00) | 69.16 (8.30) | 44.20 (6.09) | 32.49 (5.44) | 24.05 (3.49) | 19.93 (2.10) | 17.98 (1.01) |
| **RM-SORN** | 99.58 (0.24) | 99.34 (0.35) | 93.68 (5.61) | 60.32 (9.58) | 35.93 (5.81) | 22.65 (4.63) | 17.57 (1.78) | 16.91 (0.49) | 16.63 (0.38) |
| **Random** | 29.54 (7.85) | 26.74 (6.35) | 20.41 (3.28) | 19.23 (1.72) | 17.99 (1.29) | 17.11 (0.66) | 16.72 (0.40) | 16.63 (0.35) | 16.54 (0.38) |

**Markov-85 Task**

**Supplementary Table 29: Markov-85 Performance; n from -10 to -1**

| n | -10 | -9 | -8 | -7 | -6 | -5 | -4 | -3 | -2 | -1 |
|---|---|---|---|---|---|---|---|---|---|---|
| **Static** | 49.30 (2.87) | 53.75 (3.46) | 58.34 (3.27) | 63.80 (3.65) | 69.68 (3.49) | 76.44 (3.46) | 82.90 (2.87) | 89.64 (2.31) | 96.34 (1.43) | 99.83 (0.19) |
| **SORN** | 52.68 (4.13) | 57.74 (4.67) | 63.06 (4.76) | 69.33 (4.59) | 75.99 (4.62) | 82.58 (4.51) | 88.21 (3.95) | 93.33 (2.91) | 97.88 (1.38) | 99.70 (0.32) |
| **RM-SORN** | 49.58 (4.25) | 53.81 (4.37) | 60.04 (4.49) | 65.49 (4.96) | 72.74 (4.88) | 79.74 (4.18) | 86.07 (3.49) | 92.08 (2.64) | 96.68 (1.38) | 98.91 (0.47) |
| **Random** | 26.62 (3.99) | 26.54 (4.02) | 31.24 (4.99) | 30.66 (5.79) | 33.55 (6.23) | 34.33 (7.45) | 40.36 (8.97) | 35.37 (8.93) | 44.97 (10.32) | 40.53 (10.22) |

**Supplementary Table 30: Markov-85 Performance; n from 0 to 10**

| n | 0 | 1 | 2 | 3 | 4 | 5 | 6 | 7 | 8 | 9 | 10 |
|---|---|---|---|---|---|---|---|---|---|---|---|
| **Static** | 100.00 (0.00) | 86.88 (0.28) | 75.78 (0.52) | 66.42 (0.71) | 58.61 (0.81) | 52.12 (0.85) | 46.53 (0.80) | 41.73 (0.75) | 37.62 (0.74) | 34.26 (0.69) | 31.51 (0.69) |
| **SORN** | 100.00 (0.00) | 86.87 (0.30) | 75.77 (0.52) | 66.41 (0.71) | 58.60 (0.81) | 52.10 (0.85) | 46.51 (0.80) | 41.72 (0.75) | 37.61 (0.74) | 34.25 (0.68) | 31.48 (0.68) |
| **RM-SORN** | 99.59 (0.27) | 86.02 (0.57) | 74.57 (0.80) | 64.58 (1.21) | 56.35 (1.44) | 50.21 (1.36) | 45.10 (1.00) | 40.12 (0.97) | 36.05 (1.07) | 32.52 (0.96) | 29.73 (0.97) |
| **Random** | 41.22 (10.65) | 33.56 (8.91) | 38.03 (8.44) | 29.35 (6.41) | 30.74 (5.38) | 26.52 (4.90) | 25.98 (3.73) | 22.67 (3.42) | 23.39 (3.19) | 20.75 (2.53) | 21.22 (2.06) |

## Pattern Recognition Task

### Supplementary Table 31: Pattern Recognition Performance

| N | 10 | 20 | 30 | 40 | 50 | 60 | 70 | 80 | 90 | 100 |
|---|---|---|---|---|---|---|---|---|---|---|
| **Static** | 84.63 (3.91) | 88.93 (2.66) | 89.92 (2.84) | 95.40 (2.72) | 93.77 (3.38) | 96.39 (1.86) | 97.70 (1.92) | 97.06 (1.94) | 97.82 (1.93) | 94.06 (2.98) |
| **SORN** | 79.30 (4.03) | 85.87 (3.63) | 88.61 (4.98) | 90.53 (3.00) | 89.47 (4.84) | 91.83 (3.95) | 93.63 (2.64) | 93.65 (3.35) | 94.68 (2.87) | 89.61 (4.82) |
| **RM-SORN** | 87.53 (4.31) | 93.77 (4.26) | 97.48 (2.86) | 95.50 (2.15) | 97.06 (1.98) | 97.04 (2.08) | 97.09 (1.68) | 97.47 (1.72) | 96.72 (1.52) | 96.87 (2.37) |
| **Random** | 77.48 (3.72) | 78.04 (3.96) | 77.67 (3.76) | 79.10 (4.82) | 77.36 (3.13) | 78.06 (3.60) | 80.72 (4.97) | 76.85 (2.81) | 76.04 (4.69) | 76.24 (3.84) |
